# Development and Economic Evaluation of a Patient-Centered Care Model for Children With Duchenne Muscular Dystrophy: Protocol for a Quasi-Experimental Study

**DOI:** 10.2196/42491

**Published:** 2023-04-28

**Authors:** Titiksha Sirari, Renu Suthar, Amarjeet Singh, Shankar Prinja, Vishwas Gupta, Manisha Malviya, Akashdeep Singh Chauhan, Naveen Sankhyan

**Affiliations:** 1 Lovely Professional University Phagwara India; 2 Pediatric Neurology Unit, Department of Pediatrics Postgraduate Institute of Medical Education and Research Chandigarh India; 3 Department of Community Medicine School of Public Health Post Graduate Institute of Medical Education and Research Chandigarh India; 4 Community Medicine Shri Ram Murti Smarak Institute of Medical Sciences Bareilly, Uttar Pradesh India; 5 Symbiosis Centre for Management Studies Noida, Uttar Pradesh India; 6 King’s Technology Evaluation Centre King's College London London United Kingdom

**Keywords:** Duchenne muscular dystrophy, patient-centered care, disabilities, quality of life, caregivers, cost of illness, effective, treatment, policy, caregiver, pediatrics, intervention, psychological, disability

## Abstract

**Background:**

Duchenne muscular dystrophy (DMD) is a rare progressive muscular disease that primarily affects boys. A lack of comprehensive care for patients living with DMD is directly associated with a compromised quality of life (QoL) for those affected and their caregivers. This disease also has a huge economic impact on families as its treatment requires substantial direct, indirect, and informal care costs.

**Objective:**

This study presents a protocol developed to evaluate the feasibility and efficacy of a patient-centered care (PCC) model for children with DMD. The care model was designed with the aim to empower families, improve QoL, and reduce economic burden on their families.

**Methods:**

This study is planned as a quasi-experimental study that will enroll 70 consecutive families with boys (aged 5-15 years) with DMD visiting a tertiary care center. The study is being conducted in 2 phases (preintervention and postintervention phases, referred to as phase 1 and phase 2, respectively). During phase 1, the patients received routine care. The study is now in phase 2, with the intervention currently being administered. The intervention is based on the PCC model individualized by the intervention team. The model has a comprehensive DMD telecare component that includes teleconsultation as one of its key components to reduce in-person physician visits at the health facility. Teleconsultation is especially beneficial for late-ambulatory and nonambulatory patients. Data on economic burden are being collected for out-of-pocket expenses for both phases during in-person visits via telephone or messaging apps on a monthly basis. QoL data for patients and their primary caregivers are being collected at 3 time points (ie, time of enrollment, end of phase 1, and end of phase 2). Outcome measures are being assessed as changes in economic burden on families and changes in QoL scores.

**Results:**

Participant recruitment began in July 2021. The study is ongoing and expected to be completed by March 2023. The findings based on baseline data are expected to be submitted for publication in 2023.

**Conclusions:**

This paper outlines a research proposal developed to study the impact of a PCC model for patients with DMD in low- and middle-income countries (LMICs). This study is expected to provide evidence of whether a multicomponent, patient-centric intervention could reduce economic burdens on families and improve their QoL. The results of this study could guide policy makers and health professionals in India and other LMICs to facilitate a comprehensive care program for patients living with DMD. The economic impact of a rare disease is an important consideration to formulate or evaluate any health policy or intervention related to new treatments and financial support schemes.

**Trial Registration:**

Clinical Trials Registry India (ICMR-NIMS) CTRI/2021/06/034274; https://www.ctri.nic.in/Clinicaltrials/pmaindet2.php?trialid=56650

**International Registered Report Identifier (IRRID):**

PRR1-10.2196/42491

## Introduction

### Background

Duchenne muscular dystrophy (DMD) is an X-linked recessive disease with an incidence of 1 in 3500 to 6000 live male births [1]. It progresses from an ambulatory to a nonambulatory stage, with the child usually becoming wheelchair-bound between 11 and 13 years and death occurring in the late teens or early twenties [2-4]. Even with this short span of life, children with DMD face many challenges, as their progression through various transitional phases impacts not only them but also their families and society at large [5].

The progressive and irreversible damage to the muscles results in weakness, loss of ambulation, breathing issues, and cardiomyopathy. Currently, there is no definitive treatment to halt the disease’s progression. Disease management mostly revolves around symptom management and involves a multidisciplinary approach to address various systemic complications [6].

### Course of Disease

Boys with DMD usually remain asymptomatic up to 2 years of age and get diagnosed between 4 to 6 years of age. Most of the boys with DMD are limited to assisted ambulation with the help of long leg braces by the age of 10 years. Age of loss of ambulation globally is between 13 and 14 years, whereas in resource-limited settings, it is between 9 and 11 years [4]. There is no study on the prevalence or incidence of DMD in India. However, a study from southern India [7] on 275 genetically confirmed patients with DMD was conducted to study the natural course of motor milestones. Authors reported the mean age of onset of symptoms was 3.7 years, and the mean age at presentation was 8.1 years. During the follow-up period of 15 years, 155 out of 275 (56.4%) children had either become wheelchair-bound or bed-bound or died. The affected children were wheelchairs bound by a mean age of 10.4 years and bed-bound by 11.8 years. Seven boys (2.6%) died during the follow-up period at a mean age of 15.2 years.

DMD requires a multidisciplinary team approach for holistic management. This includes diagnostic services, physical therapy, orthotics, respiratory therapy, corrective orthopedic surgery, ventilation, feeding support, and speech therapy. Muscle degeneration can be slowed down with the use of corticosteroids, while antibiotics may be needed for respiratory infections [8-11]. Occupational therapy, assistive devices, and a wheelchair are also beneficial to the children. Some patients may need a pacemaker for cardiac abnormalities and assisted ventilation for respiratory muscle weakness. Patients diagnosed with DMD should expect to make a successful transition to adulthood, which includes education, health care, and social support to transition smoothly from adolescence to adulthood [12,13]. 

The DMD Care Considerations Working Group identified eleven components of care: (1) diagnosis, (2) neuromuscular management, (3) rehabilitation management, (4) gastrointestinal and nutritional management, (5) respiratory management, (6) cardiac management, (7) orthopedic and surgical management, (8) psychosocial management, (9) primary care and emergency management, (10) endocrine management (including growth, puberty, adrenal insufficiency, and bone health), and (11) transitions of care across the life span. The mainstay of management is still limited to physiotherapy and glucocorticoids therapy, which help in delaying loss of ambulation, an important milestone of the disease [6,14]. Treatment modalities also have changed, especially with the advent of exon-skipping therapy and gene editing tools. However, these new modalities of treatment have not made a significant clinical impact yet [15].

### Empowering Patients and Caregivers

Globally, we are witnessing a new paradigm shift to resolve problems from a mechanistic or Newtonian worldview to a holistic view. This approach involves human values, creativity, and evolution, and it has affected the approach toward understanding human health. Nevertheless, the reductionistic approach dominates the imagination of the scientific fraternity [16]. The biomedical or reductionistic approach sees patients as an isolated problem, whereas we need to understand that a holistic approach (ie, social, economic, sanitary, environmental, and political commitment) is needed to manage a disease.

An inclusive paradigm has two major components: (1) self-empowerment of the patient and (2) holism. Empowering patients involves developing skills, control over resources, ensuring autonomy in decision-making, and taking ownership of their health [17,18].

Empowering patients through an effective health care delivery model has the potential to improve the cost-effectiveness of care, especially for people affected by long-term conditions [19]. Patient-centered care (PCC) increases adherence to disease management, reduces loss to follow-up in the health care system, and, in turn, reduces morbidity and improves quality of life (QoL) for patients [17].

### QoL of Children Living With DMD

With the advancement in medical management, patients with DMD are reported to live longer up to the third decade of life. These require more years to live with functional disability and more years of caregiving. Past studies have reported variable QoL among patients with DMD, with some studies showing poor QoL and others showing no difference between healthy children and those with DMD. Being able to participate in various activities of daily living, like personal care, mobility, social relationships, education, recreation and leisure, spirituality, and community life, are outcomes of health. Physical disability substantially decreases participation and consequently impairs QoL [18]. Studies have reported that only physical participation levels were much lower in patients with DMD, and other participation levels were almost the same.

Bendixen et al [20] compared participation in life activities and perceived QoL between younger and adolescent boys with DMD and age-matched controls. No differences in perceived QoL were observed between the participants with DMD and the healthy controls, but the older adolescent participants with DMD had significantly lower QoL subscales of physical, social, and total composite scores. de Moura and colleagues [21] studied the relationship between physical dependence and QoL of patients with DMD and the QoL and burden of caregivers. Lower QoL was observed in caregivers with increased age and the burden of caregiving. In a review, Landfeldt et al found [22] the informal care of patients with DMD was associated with a substantial burden, and they recommended further research to better understand the relationship between clinical implications and progression of disease and caregiver burden. A cross-sectional study by Jackson et al [23] examined mothers’ demographic characteristics and their sons’ QoL-related factors to identify which ones contribute to poorer health-related QoL and greater emotional distress. Mothers’ perception of their sons’ health-related QoL is a significant predictor of their own health-related QoL and emotional distress. In turn, mothers’ physical and emotional health influences their social activities. Social activities are important to manage stress among caregiving parents. QoL is a measure used to obtain additional information on the effectiveness of clinical measures, the quality of services they need for health care, the effectiveness of the interventions, and cost-utility analyses. Change in QoL reflects how much a patient is satisfied with his or her treatment or the system of health care delivery [20,24,25].

### Economic Burden of Disease

Previous studies have revealed that DMD also has a substantial economic burden on families. Studies have shown that this burden increases markedly with disease progression; however, no such study has been conducted in the Southeast Asian region [22,26].

Health care delivery systems frequently use patient engagement to reduce expenditure and improve health outcomes. When analyzing the health care delivery system in the state of Minnesota, Hibbard et al [27] concluded that patients who were less actively engaged in their treatment had 8 times higher health care costs in the first year, and this was significantly different from patients who were actively engaged in their treatment. Another review by Hibbard and Green [28] on patient engagement concluded that various tailor-made interventions to increase patient involvement in their disease significantly improved health outcomes. Nevertheless, very few studies comment on costs related to disease. Proper allocation of resources is most important for any institution to have sustainable development, and this applies to the health care system as well. Cost-of-illness studies can provide important information to policy makers at both the micro (eg, cost-effectiveness of new interventions) and macro level (eg, fund allocation for further research and prioritization of disease for preventive interventions) [13,29-31].

The Indian Ministry of Health and Family Welfare has begun to address this issue related to rare diseases, as reflected in its 2017 National Health Policy. The Indian government approved the separate National Rare Disease Policy in 2021. This policy recommends a focusing on indigenous research and local production of medicines, searching for low-cost treatments for rare diseases, and screening and early detection. Financial support is sought to be given to the rare diseases classified as Group 1 in the policy under the umbrella of Rashtriya Arogya Nidhi or Pradhan Mantri Jan Arogya Yojana. However, DMD is classified under Group 3, described as “diseases for which definitive treatment is available but challenges are to make optimal patient selection for benefit, very high cost and lifelong therapy” [32]. The policy identified 8 tertiary care centers as Centers of Excellence for managing rare diseases. The hospital-based National Registry of Rare Diseases was created in these centers to ascertain the exact burden of rare diseases.

Accordingly, this study aims to estimate the economic burden incurred by the families of children with DMD seeking care at a government hospital. Next, we aim to develop a PCC model (intervention) to build the capacity of affected families to cope with DMD, thus reducing the financial burden on families. Finally, we aim to compare the impact of intervention with routine care on QoL (caregiver and patient) and economic burden after 6 months of intervention.

## Methods

### Study Design

This is a longitudinal quasi-experimental study that will be conducted in 2 phases. It is important to note that within the scope of this study, it is difficult to achieve a controlled experimental design. There is no randomization, and the same group of subjects are to be compared before and after the intervention. The experimental condition may be systematically different across the time frame. Considering this limitation, we chose a quasi-experimental design for this study. [33]

Phase 1 of the study is observational, and no intervention is administered. Participants (previously diagnosed and under follow-up as well as new patients) were recruited prospectively from pediatric neurology clinic, department of Pediatrics, Advanced Pediatrics Center, Post Graduate Institute of Medical Education & Research (PGIMER), Chandigarh, India. Detailed information about the study was provided to the children and their caregivers. After providing consent to participate in the study, the primary caregivers were interviewed for demographic information, clinical information, and information related to their economic status and financial burden of disease caused by DMD. The QoL of patients and caregivers was assessed with the EuroQoL 5-Dimension 5-Level (EQ-5D-5L) scale and the World Health Organization Quality-of-Life Scale (WHOQOL-BREF), respectively. After recruitment into the study, the patients were followed up for 6 months. Routine treatment as per institutional norms was given during this period. The primary caregiver was contacted via telephone monthly for data collection on economic burden. After the 6 months, the participants entered into the next phase (ie, phase 2).

Parallelly, during phase 1, the PCC intervention model was developed. The rationale behind developing the care model was to provide basic information about DMD and treatment options to the patients and their parents or caregivers so that they could participate effectively in the process of obtaining comprehensive care in a resource-limited setting. The intervention includes information about the disease, an instructional booklet, and a workbook to encourage compliance. This study also includes a teleconsultation with pediatric neurologist and the rehabilitation team. The sessions are designed to facilitate caregivers in decision-making through active participation in every stage of the disease. This set of interventions are collectively referred to as the Comprehensive DMD-Telecare Model.

**Figure 1:**
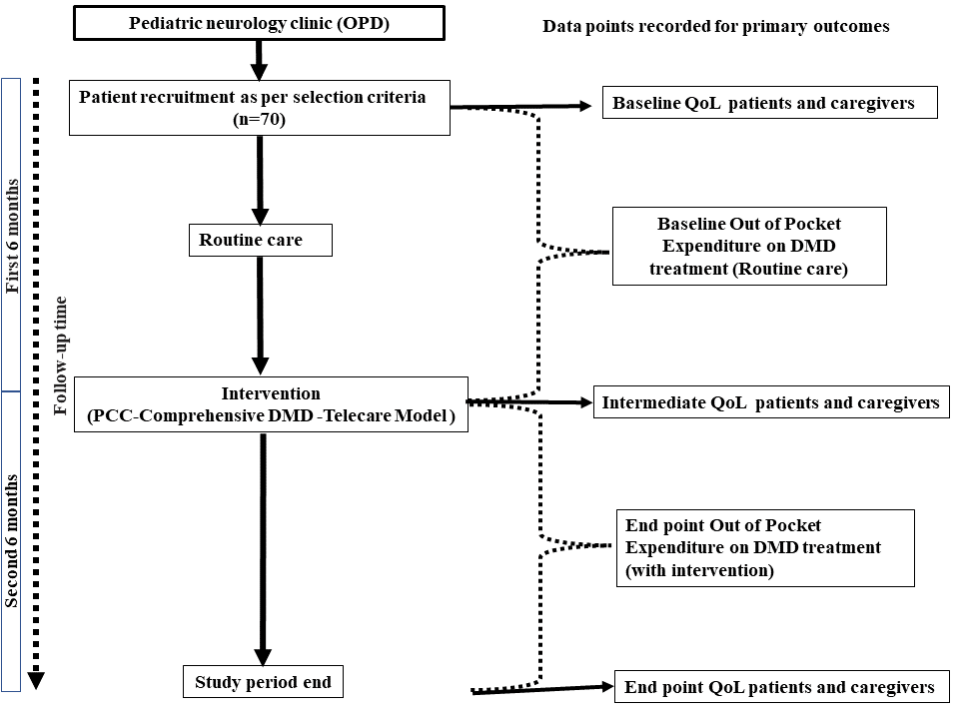
Overview of study design. DMD: Duchenne muscular dystrophy; OPD: Outpatient Department; PCC: patient-centered care; QoL: quality of life.

Phase 2 is the interventional phase of the study, which commences once the patient has completed phase 1 (ie, the first 6 months of the study). During phase 2, patients and their parents or caregivers are given a set of interventions developed during phase 1. The patients are followed up prospectively for the subsequent 6 months (Figure 1), and data on economic burden are recorded telephonically on monthly basis. Children are visiting the health facility just twice during the follow-up period (ie, at the beginning and at the end of phase 2). This reduced the number of in-person visits the patients had to make to the health facility.

### Ethics Approval

Approval for this study was granted from the ethics committees of PGIMER (IEC-01/2021-1884) and Lovey Professional University (LPU/IEC/2021/01/24). The patients and their caregivers were informed about the study and provided consent before recruitment. Information collected from the patients and their caregivers will be anonymized. The study protocol was registered with the Clinical Trials Registry India (CTRI/2021/06/034274).

### Setting and Sample Size Considerations

Patients were recruited prospectively in a consecutive manner from the Pediatric Neurology Clinic of the Advanced Pediatric Center at PGIMER in Chandigarh, India. This institute caters to approximately 7 north Indian states, namely, Punjab, Haryana, Himachal Pradesh, Jammu and Kashmir, Uttarakhand, Bihar, the northwestern part of Uttar Pradesh, and the Union Territory of Chandigarh.

The sample size estimation for the primary outcome was in accordance to changes in patients’ health-related QoL (HRQOL) questionnaire scores. The QoL scores were assessed in the observational phase (0-6 months) and compared with the QoL scores in the intervention phase (7-12 months). A clinical meaningful difference based on the minimum important difference from HRQOL was 0.1 [34], along with an SD of 0.2 [35] and an allocation ratio of 1:1. The estimated sample for the study was 126 (63 patient in each phase) to achieve a level of significance of 95% CI at a power of 80%, accounting for a 10% dropout rate. The total sample size was 140, with 70 patients in each phase.

### Study Population

The study population comprises families or caregivers with boys aged between 5 and 15 years who were genetically diagnosed with DMD and are seeking consultation at the pediatric neurology clinic of the government tertiary care center. Caregivers defined as the persons who look after the patients in the hospital or at home (parents or family members). This excludes hospital staff and paid workers hired by the family.

### Selection Criteria

Boys with DMD presenting to the Pediatric Neurology Clinic were screened for enrollment in the study. The selection of participants was carried out by a pediatric neurologist. Those who fulfilled the inclusion and exclusion criteria were included in the study after providing informed consent. Textbox 1 lists the inclusion and exclusion criteria for this study.

**Inclusion and exclusion criteria for this study.**

**Inclusion criteria**

- Families with boys aged 5 between 15 years who have Duchenne muscular dystrophy (DMD) or Becker muscular dystrophy (BMD) and display clinical symptoms and creatine kinase (CK) levels elevated 10 times or more the normal level
- Laboratory genetic confirmation of DMD or BMD by multiplex ligation-dependent probe amplification (MLPA), polymerase chain reaction (PCR), next-generation sequencing (NGS), or muscle biopsy (showing absence of dystrophin expression on immunohistochemistry)
- Caregivers of the patients with who have access to a smartphone and internet connection

**Exclusion criteria**

- Caregivers and patients who are unwilling to participate or unable to comply with the study protocol and visits
- Caregivers and patients who did not provide consent to participate

### Intervention Program

#### Routine Care

Boys with clinical symptoms suggestive of DMD were first consulted and evaluated in the pediatric neurology clinic. After detail clinical investigations, a treatment plan for the patient was decided as per the institutional protocol for the management of muscular dystrophies and discussed with the patient. The patient was required to visit the health facility routinely once every 3 months. For multispecialty care, patients visited different subspecialists as required. Physiotherapy and orthosis appointments, along with visits to specialty care units, increase hospital visits, often leading to confusion, treatment nonadherence, and loss to follow-up. The components of routine management are given in Textbox 2.

**The components of routine management as per institutional protocol (standard operating care).**

1. Confirm diagnosis: clinical examination, investigations, confirmation of diagnosis with genetics, and muscle biopsy
2. Family counseling
3. Baseline assessment of motor attributes with the 6-Minute Walk Distance Test, Time Function Test, and Vignos and Brooke scales
4. Baseline cardiac, respiratory assessment, spine, bone health, and endocrine evaluation
5. Oral supplementals of calcium and vitamin D
6. Nighttime ankle-foot orthosis
7. Physical therapy muscles strengthening and stretches to reduce contractures
8. Vaccination (immunization)
9. Steroids as per standard guidelines
10. Dietary advice
11. Advice on play and leisure
12. Three monthly clinical check-ups for disease progression, monitoring for weight, height, blood pressure, ophthalmic evaluation, bone health, and steroid side effects (these visits may be more as per disease progression requirement).
13. Prenatal diagnosis, carrier screening, and genetic counseling

#### PCC: Comprehensive DMD-Telecare Model

The rationale behind developing the PCC model for patients with DMD was to (1) provide basic information to the patient and their parents or caregivers so that they can effectively understand the disease and participate in the process of obtaining comprehensive care in a resource-limited setting and (2) create awareness about the importance of managing the disease not only pharmacologically but also by improving QoL.

**Figure 2:**
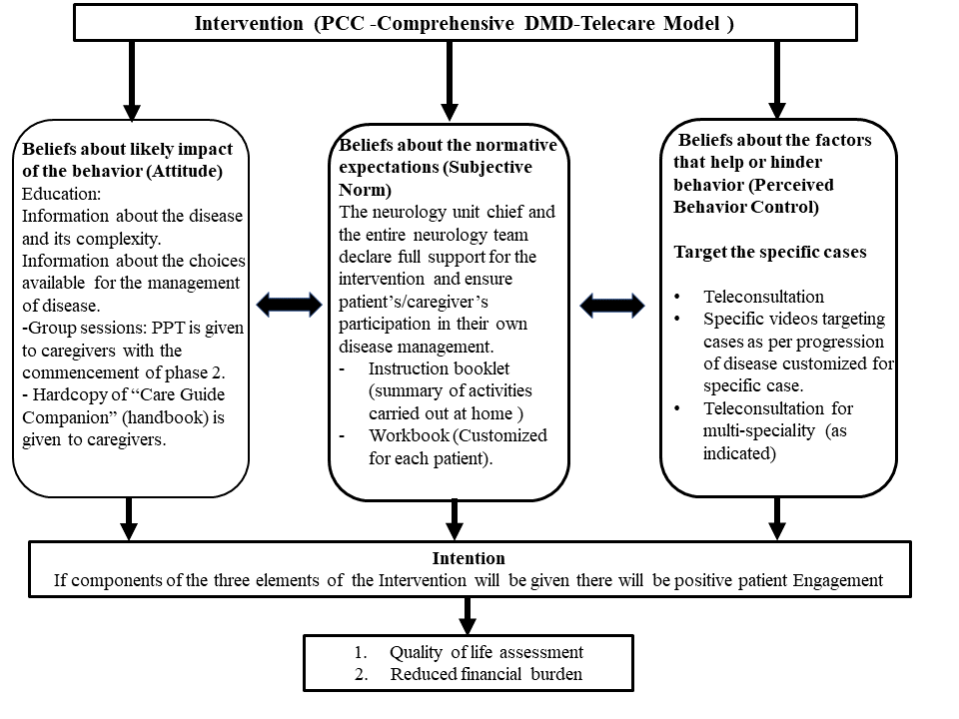
Overview of patient-centered care (PCC): intervention model. DMD: Duchenne muscular dystrophy; PPT: PowerPoint presentation.

After a systematic review of literature, the intervention designed was based on the Theory of Planned Behavior and tailored to each stage of the study [36]. The conceptual model of PCC is given in Figure 2. The telecare component was added to the model considering the challenges that patients and their caregivers experience when having to travel to the heath care facility frequently, especially in low- and middle-income countries (LMICs). The components of the intervention are provided in Table 1.

The purpose of the intervention is to reduce the number of in-person visits the patient has to make to the clinic. The planned physical follow-up to the health facility during phase 2 is at 6 months. Families are called for physical follow-up only if indicated and instructed by the clinician within 6 months. These visits are recorded.

Protocol material for the Comprehensive DMD-Telecare Model is circulated among pediatricians and pediatric physiotherapists working at the Pediatric Neurology Clinic, School of Public Health experts at PGIMER, and caregivers or parents.

A preliminary version of this package was circulated among specialists for consensus validation and modified according to the feedback received. Thereafter, it was pilot tested among 5 patients and their caregivers. Further corrections were made as needed. Textbox 3 lists the outcomes of the study.

**Table 1:** Components of the intervention.

Component	Description
Informational session about the disease	Information sessions are carried out via PowerPoint presentation, and a “Care Guide Companion” booklet is provided (both hard copy and digital). The booklet is designed to helping patients and their caregivers participate in decision-making for their treatment. The components of the booklet include (1) basic information related to DMD^a^, (2) management of DMD, (3) role of the care provider team, and (4) support groups to help people living with DMD.
Instructions and checklist	Instructions are given to the patients and their caregivers to make them understand all the activities that will be carried out once they enter phase 2 of the study. Audiovisual media and images containing instructions for stretches or physiotherapy and physical activity are provided to the family.
Compliance diary	The patient is expected to comply with the prescribed procedures and medications. A customized daily planner is introduced to each patient in phase 2 to increase their adherence to the protocol. This contains periodic monitoring of weight, height, blood pressure, daily medication (eg, steroids, ACE^b^ inhibitors, calcium, and vitamin D), and daily exercise routine. Some clinical evaluations are expected to be performed at home (eg, Gower sign, Vignos score, Brookes scores, etc) which are monitored by the research team over the phone.
Teleconsultation	A total of 3 monthly video teleconsultations are given to the patients by clinicians to provide diagnostic or therapeutic advice electronically. The patient does not have to visit the clinic in person. This is done through: (1) review videos that the family shares with the care team regarding the patient’s present state of illness. These videos are recordings of physical activities that the patient can perform at home, which are share with the team for evaluation purposes; and (2) customized videos: As per the clinical requirements, customized videos are shared with patients to address any additional needs.
Multidisciplinary teleconsultation	Not all subspecialists are needed at all ages or stages, but they must be accessible if necessary.

^a^DMD: Duchenne muscular dystrophy.

^b^ACE: Angiotensin-converting enzyme.

**Study outcomes.**

**Primary outcomes**

1. Difference in quality of life (QoL) with and without the intervention
2. Difference in out-of-pocket (OOP) expenses incurred by patients and their caregivers with and without the intervention
3. Development of patient-centered care (PCC) model known as the Comprehensive Duchenne muscular dystrophy (DMD)-Telecare Model

**Primary outcome measures**

1. Change in QoL mean scores in phase 1 (0-6 months) and phase 2 (7-12 months) of the study
2. Change in mean OOP expenses incurred by the patients and their caregivers with and without the intervention

**Secondary outcomes**

1. Socioeconomic determinants of primary outcomes.
2. Feasibility of follow-up via telephone
3. Pattern of health care utilization for different stages of DMD
4. Neuromuscular QoL in the pediatric age group
5. Opinions and experiences related to the challenges faced by stakeholders during the management of DMD (noted verbatim)

**Secondary outcome measures**

1. Demographic data and socioeconomic status in terms of means and percentages
2. Compliance with telephone follow-up and satisfaction index on Likert scale
3. Mean score of neuromuscular QoL in the pediatric age group
4. Thematic analysis of the qualitative data

### Data Collection

At the time of recruitment, primary caregivers are interviewed on their social and demographic characteristics and QoL. Further, after the initiation of phase 1, these caregivers are contacted again prospectively to assess the economic burdens they face by collecting data on out-of-pocket (OOP) expenses incurred during the treatment of DMD. The data on economic burden include information on household consumption expenditure, patient-level expenses incurred on diagnosis and treatment related to DMD, and coping mechanisms for dealing with DMD. A pretested semistructured schedule, adapted from previous studies in similar settings, is used to interview the patients [37-39]. Cost-of-illness (COI) analysis is carried out to assess OOP expenses, which are further classified as direct and indirect expenses [40]. Expenditure on the diagnosis, drugs or consumables, hospitalization, user fee, or procedure fee is considered direct health care expenditure. Transportation, boarding, loading, and food are taken as direct non–health care expenditures. Wage or income loss by the accompanying caregivers during the period of treatment is considered an indirect expenditure.

Information on economic burden is gathered from the caregivers throughout the follow-up period of 12 months. Data are collected during their physical visits as well as telephonically every month.

HRQOL of the patients and their caregivers are gathered at the baseline and then at the completion of phases 1 and 2. QoL of caregivers is also recorded at the 3 data points (baseline, end of phase 1, and end of phase 2). The study design is further explained in Figure 1.

### Data Management and Analysis

Primary data are first entered in Epi Info software (version 7; Centers for Disease Control and Prevention). Data will be analyzed using both Epi Info 7 and SPSS software (version 23; IBM Corp). Mean OOP expenditure incurred due to the treatment modalities will be calculated. The OOP expenditure will be reported in Indian rupees. For international comparison, expenses will be converted to US dollars using an appropriate conversion rate. Indirect expenditure will be calculated for the duration of follow-up treatment using a human capital approach by assessing the wage loss of the caregivers [41]. Financial risk will be assessed in terms of catastrophic health expenditure and distress financing. An expenditure on DMD treatment that exceeds the threshold of 40% of nonfood household consumption expenditure is considered a catastrophic health care expenditure [42,43]. Households that had to either borrow money (with or without interest) or sold their assets (such as land, home, cattle, etc) to cope with DMD-related expenditure will be classified as having faced distress financing [38,44,45]. Multiple logistic regression analysis will be performed to examine the risk of catastrophic health expenditure and distress financing with covariates, including age, income status, treatment modality, education, locality, and stage at the time of diagnosis. Sensitivity analysis will be carried out to assess the prevalence of catastrophic expenditure at varying cutoff levels (ie, 20%-50%).

The HRQOL of the patients will be assessed by the EQ-5D-5L tool. This tool comprises the EQ-5D descriptive system and a visual analog scale (EQ VAS). The EQ-5D-5L descriptive system has 5 dimensions (ie, mobility, self-care, usual activities, pain or discomfort, and anxiety or depression). Each of these dimensions has a response in the form of 5 levels of perceived problems: no problems (level 1), slight problems (level 2), moderate problems (level 3), severe problems (level 4), and extreme problems (level 5). Thus, a unique health state is defined by combining 1 level from each of the 5 dimensions. The EQ VAS records the patient’s self-rated health on a VAS, where the end points are labeled as “The best health you can imagine” and “The worst health you can imagine.” The analysis of the QoL utility scores based on the EQ-5D-5L tool will be done as recommended in the user guide. Each of the patient’s condition or state at a particular time period will be referred to in terms of a 5-digit code. For example, state 11111 implies no problems on any of the 5 dimensions, while state 55555 indicates extreme discomfort in each of the dimensions. Each of these states are further converted into a single index utility value based on the recently generated tariff value set for the Indian population [46,47].

As mentioned earlier in this paper, the self-administered WHOQOL-BREF tool is used to assess the QoL of caregivers. This tool has 26 items describing 4 QoL domains, namely, physical health (7 items), psychological health (6 items), social relationships (3 items), and environmental health (8 items). It also contains items for general QoL and overall health (2 items). The response to each item is recorded in 5 levels and scored from 1 to 5. The raw scores will be converted into transformed scores ranging from 4 to 20. Later, these transformed scores will be converted into main domain scores ranging from 0 to 100, with higher scores indicating better QoL [48].

Correlations of HRQOL in children and QoL of caregivers in all 4 domains at the baseline will be assessed with socioeconomic status (SES), COI, and also with each other. Multiple linear regression analysis will be conducted to address the confounders (eg, age at baseline, family structure, type of disease, family history, family structure, education, and SES).

Economic burden and QOL data will be analyzed as per the study objective, and comparative analysis will be done to justify the hypothesis. Percentage, mean, and SD values will be recorded, and the chi-square test, paired *t* test, and Mann-Whitney *U* test will be carried out for statistical analysis. SPSS software (IBM Corp) will be used for the analyses.

## Results

Study enrollment was completed in March 2022 with a total of 100 children with DMD and their caregivers (125% of the target). All 100 patients and their caregivers were followed up for 6 months in phase 1 of the study, which was completed in July 2022. The intervention was administered to 66 patients and their caregivers (94.3% of the target population) in phase 2. The participants were further followed for the subsequent 6 months, and phase 2 ended in December 2022. We are in the process of data entry and data cleaning. Initial data show that the children range in age between 5 and 15 years at enrollment, with a mean age of 8.3 (SD 1.9) years. Most of the children (56/100, 56%) enrolled in this study are in the late ambulatory disease (stage 3), and only 1 (1%) child was in a late nonambulatory stage at the time of enrollment. However, these dynamics changed swiftly with the progression of the disease.

Primary caregivers enrolled (n=100) at the baseline in the study were mostly mothers (58/100, 58%), with fathers comprising 38% (38/100) of the caregivers. Only 57% (57/100) of caregivers had some knowledge about DMD before enrollment. About 75% of the caregivers were educated more than high school, and 8.2% of them did not receive any formal education. More than half (51/100, 51%) the patients were had upper-middle SES, and (3/100, 3%) had lower SES. Because this study is only in the data entry and data cleaning phase, no impact data are available to report on the study objectives, aims, and hypotheses. The findings are expected to be published in 2024.

## Discussion

This quasi-experimental study is expected to give baseline data about the economic burden faced by families who have children living with DMD for the first time in the Southeast Asian region. The PCC model (intervention) developed during the study period is based on individual needs and is a part of standard operating care (SOC). The intervention is mainly focused toward nonambulatory patients, as it is difficult for them to travel to health facilities. The health facility is actively approaching these patients to streamline their disease management and reduce loss to follow-up. Later, we also expect to evaluate whether this PCC model (intervention) can help reduce the financial burden faced by caregivers of children with DMD and help increase the QOL of both patients and their caregivers. The intervention has been designed considering the needs of all stakeholders (patients, parents or caregivers, and health care providers) by conducting group discussions, which are essential for the success of any intervention. The effectiveness of the program will be evaluated by collecting postintervention data.

The care required for a rare disease like DMD is not the same as usual medical care. For rare diseases, the patient- or caregiver-physician relationship grows with time, along with an evolution in the knowledge of the disease and its management strategies. As the disease outcomes and management are formative, the health care provider must inform and engage patients and their caregivers as an interactive partner to encourage compliance and adherence to the SOC. Most of the time, treatment seeking by the patients is limited by scarce knowledge about the disease, lack of availability of appropriate multidisciplinary health care, and limited access to treatment modalities [49]. The involvement of multiple disciplines is often overlooked by the health care sector. In addition to medical management, self-care, as well as caregiving by the patients’ parents, is very important. Patient engagement when managing a disease is very promising, as the patient takes ownership of his or her health. PCC is one such intervention that ensures patient involvement. Patient or family engagement improves health outcomes and reduces OOP health expenditures. Patient engagement, health outcomes, and health expenditure depend on the health care delivery system of the geographical region. The way that the health care delivery system functions depends on preferences, values, cultural traditions, and socioeconomic conditions in a given region [50].

The model will have more geographical coverage through teleconsultation, especially considering the COVID-19 era where travelling is restricted. Once the model is proven successful, it can be replicated for managing other chronic rare diseases. Nevertheless, this study has certain limitations, as out study design is noncontrolled, there is no randomization, and a single group is compared before and after the intervention. The experimental conditions may be systematically different across the study time frame. Our study aims to analyze economic burdens among ambulatory patients with DMD visiting the health care facility and are mostly within the age group of 5 to 15 years. However, previous studies have shown that that the maximum financial burden is faced in the later stages of disease (Stage 5, late nonambulatory), but this group is not covered in this study.

There have been studies conducted on QOL and health expenditure for DMD in Europe and North America, but no such study exists from the Asian region. Therefore, the results from this study will contribute to new scientific evidence on effectiveness of PCC for rare diseases in LMICs, where there is a paucity of research on this topic. The study has public health implications in generating evidence on the effectiveness of the intervention by considering patients’ involvement in managing DMD. This can guide policy makers on formulating policies or guidelines for a more patient-centric program to manage a chronic, lifelong rare disease not only in India but also in other LMICs as well.

## Abbreviations

COI: cost of illness
DMD: Duchenne muscular dystrophy
EQ-5D 5L: EuroQoL 5-Dimension questionnaire
HRQOL: health-related quality of life
LMIC: low- and middle-income country
OOP: out of pocket
PCC: patient-centered care
PGIMER: Post Graduate Institute of Medical Education & Research
QoL: quality of life
SES: socioeconomic status
SOC: standard operating care
VAS: visual analog scale
WHOQOL-BREF: World Health Organization Quality-of-Life
